# Unimodal productivity–biodiversity relationship along the gradient of multidimensional resources across Chinese grasslands

**DOI:** 10.1093/nsr/nwac165

**Published:** 2022-08-18

**Authors:** Yanfen Wang, Jianqing Du, Zhe Pang, Yali Liu, Kai Xue, Yann Hautier, Biao Zhang, Li Tang, Lili Jiang, Baoming Ji, Xingliang Xu, Jing Zhang, Ronghai Hu, Shutong Zhou, Fang Wang, Rongxiao Che, Di Wang, Chaoting Zhou, Xiaoyong Cui, Nico Eisenhauer, Yanbin Hao

**Affiliations:** College of Resources and Environment, University of Chinese Academy of Sciences, Beijing100049, China; State Key Laboratory of Tibetan Plateau Earth System, Environment and Resources, Chinese Academy of Sciences, Beijing100101, China; College of Resources and Environment, University of Chinese Academy of Sciences, Beijing100049, China; Beijing Yanshan Earth Critical Zone National Research Station, University of Chinese Academy of Sciences, Beijing101408, China; College of Resources and Environment, University of Chinese Academy of Sciences, Beijing100049, China; School of Grassland Science, Beijing Forestry University, Beijing100083, China; College of Resources and Environment, University of Chinese Academy of Sciences, Beijing100049, China; Beijing Yanshan Earth Critical Zone National Research Station, University of Chinese Academy of Sciences, Beijing101408, China; Ecology and Biodiversity Group, Department of Biology, Utrecht University, Utrecht3584CH, The Netherlands; College of Resources and Environment, University of Chinese Academy of Sciences, Beijing100049, China; College of Resources and Environment, University of Chinese Academy of Sciences, Beijing100049, China; State Key Laboratory of Tibetan Plateau Earth System, Environment and Resources, Chinese Academy of Sciences, Beijing100101, China; School of Grassland Science, Beijing Forestry University, Beijing100083, China; Key Laboratory of Ecosystem Network Observation and Modeling, Institute of Geographic Sciences and Natural Resources Research, Chinese Academy of Sciences, Beijing100101, China; School of Grassland Science, Beijing Forestry University, Beijing100083, China; College of Resources and Environment, University of Chinese Academy of Sciences, Beijing100049, China; Beijing Yanshan Earth Critical Zone National Research Station, University of Chinese Academy of Sciences, Beijing101408, China; College of Resources and Environment, University of Chinese Academy of Sciences, Beijing100049, China; College of Life Sciences, University of Chinese Academy of Sciences, Beijing100049, China; School of Ecology and Environmental Sciences, Yunnan University, Kunming650091, China; Institute of Biophysics, Chinese Academy of Sciences, Beijing100101, China; Department of Medicine, New York University Grossman School of Medicine, New York, NY 10016, USA; Beijing Yanshan Earth Critical Zone National Research Station, University of Chinese Academy of Sciences, Beijing101408, China; College of Life Sciences, University of Chinese Academy of Sciences, Beijing100049, China; German Centre for Integrative Biodiversity Research (iDiv), Leipzig04103, Germany; Institute of Biology, Leipzig University, Leipzig04103, Germany; Beijing Yanshan Earth Critical Zone National Research Station, University of Chinese Academy of Sciences, Beijing101408, China; College of Life Sciences, University of Chinese Academy of Sciences, Beijing100049, China

**Keywords:** productivity–biodiversity relationship, resource diversity, plant strategies, grassland

## Abstract

Resources can affect plant productivity and biodiversity simultaneously and thus are key drivers of their relationships in addition to plant–plant interactions. However, most previous studies only focused on a single resource while neglecting the nature of resource multidimensionality. Here we integrated four essential resources for plant growth into a single metric of resource diversity (RD) to investigate its effects on the productivity–biodiversity relationship (PBR) across Chinese grasslands. Results showed that habitats differing in RD have different PBRs—positive in low-resource habitats, but neutral in medium- and high-resource ones—while collectively, a weak positive PBR was observed. However, when excluding direct effects of RD on productivity and biodiversity, the PBR in high-resource habitats became negative, which leads to a unimodal instead of a positive PBR along the RD gradient. By integrating resource effects and changing plant–plant interactions into a unified framework with the RD gradient, our work contributes to uncovering underlying mechanisms for inconsistent PBRs at large scales.

## Teaser

When the multidimensional resource gradient was considered, the productivity-biodiversity relationship across Chinese grasslands was revealed to be unimodal rather than positive linear or irrelevant which were suggested by previous studies.

## INTRODUCTION

The relationship between plant productivity and biodiversity is a central topic in ecology, but has remained controversial in past decades [1–6]. One hotspot of debate is whether a positive or unimodal productivity–biodiversity relationship exists at large scales (i.e. regional and global scales) [1,2,5–8]. Many studies suggested that inconsistent productivity–biodiversity relationships probably stem from environmental variables that drive biodiversity and productivity simultaneously [1,9]. This is particularly the case for resources, which strongly affect both plant productivity [10] and biodiversity [11]. However, previous studies on the shape of productivity–biodiversity relationships have mostly focused on a certain type of resource, e.g. climatic resources [12,13], while neglecting the multidimensionality of plant resources. As such, controversial conclusions might be presented at large scales across habitats, where different resources do not always match with each other (e.g. nutrient-rich habitats are found in water-deficient sites).

The neglect of the multidimensionality of resources is probably partly due to the challenge of quantifying it [14]. Specifically, the relative importance of different resources to plant communities can be habitat dependent, leading to difficulties in establishing comparable resource gradients across various habitats in the field. Globally, productivity and biodiversity of grassland are both directly affected by climatic resources such as water and energy [9,15–18], and indirectly mediated by their influences on soil fertility [19–22]. However, soil nutrients can also be important at smaller scales [23]. Thus, controversial results of resource effects on plant communities are expected, as the resources investigated in each study are not always the same. For instance, elevated precipitation can increase plant diversity within certain boundary conditions [12,24], while increasing soil nutrient abundance usually decreases plant diversity [11,23,25]. The types of nutrient limitations may also affect the productivity–biodiversity relationship [26], and therefore, focusing on a single resource gradient might be insufficient to explore the effects of resources on plant communities. Moreover, plant communities are shaped by multiple resource limitations [10,23,27–29]. Co-limiting resources may behave as a single collective index [27], as a smooth transition from one type of limitation to another is assumed [28]. Therefore, an index that captures the multidimensionality of resources, the analogy to multifunctionality [30] and multidiversity indices [31], may help to reveal patterns and processes linking productivity and biodiversity across habitats or ecosystems, especially at large spatial scales (i.e. regional scales).

The multidimensionality of resources should contain two aspects: the average abundance of each resource (similar to plant species richness (SR)) and the evenness among them (similar to plant evenness) [32]. In contrast to plant SR, resource richness (RR) is not the total number of all necessary resources, but the overall quantity of the important resources, since most plant species have similar resource demands [33]. However, plant communities may not benefit from the high average abundance of each resource that is enhanced by only a few resources, while other resources become limiting factors. Thus, the evenness among the abundance of all resources (resource evenness (RE)) is also important to reduce the potential overestimation of resource abundance that may result from using RR alone. RE represents the ratio of all essential resources in a given habitat and is highest when a plant community is simultaneously limited by all essential resources. The ecological meaning of RE is likely an expression of the resource-related niche dimensionality [23,34,35]. Therefore, the multidimensional resource abundance should integrate both the average abundance of all resources and the evenness among them, which can be used to quantify how many resources a habitat can provide for plant communities. As such, high resource abundance signifies that all essential resources at a given habitat are abundant, while low resource abundance signifies that all essential resources are extremely low at a given habitat; and in between are habitats with moderate abundances of all resources, or habitats with low abundances for most resources but high abundances for a few others (unevenness or resource inequity).

**Figure 1.**
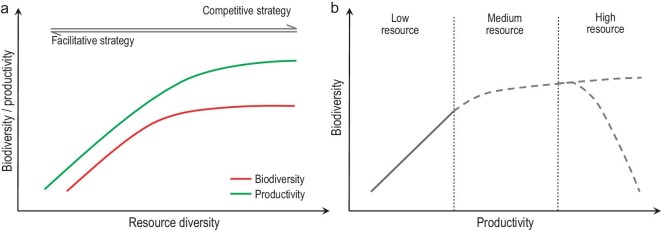
Outline of the RD hypothesis. (a) Theoretical effect of RD on productivity and biodiversity. A plateauing trend is assumed for both plant diversity and productivity, whereas the peak of diversity appears earlier than productivity. (b) Theoretical relationships between plant diversity and productivity along the resource gradient; dashed lines represent uncertain relationships. In low-resource habitats, both biotic (plant strategies) and abiotic resource effects lead to a positive productivity–biodiversity relationship. In medium-resource habitats, the biotic effect is neutral or inconsistent, and a weak positive productivity–biodiversity relationship may exist due to the abiotic resource effects. In high-resource habitats, biotic and abiotic resource effects lead to opposing relationships, resulting in either a plateauing or a negative productivity–biodiversity relationship depending on the relative effect size between the interspecific competition and the resource effect.

Based on the multidimensional resource gradient, this study aims to clarify the various effects of resources on the interactions between productivity and biodiversity. Resources can affect the productivity–biodiversity relationship through two pathways: one is a direct effect on productivity and biodiversity, while the other is through their influence on plant–plant interactions (Fig. 1a). In low-resource habitats (i.e. desert grassland), high stress levels limit the number of species that can survive [2]. Therefore, increasing resources may or may not increase plant diversity, but will likely increase plant productivity, as the species surviving such conditions can efficiently utilize the elevated resources [36,37]. As a result, in such low-resource habitats, a positive productivity–biodiversity relationship is expected due to (i) complementary effects of coexisting plant species under stressful environments [13,38]; (ii) increased productivity that enhances biodiversity from a probability point of view [39–41]; or (iii) a synergy of both. In medium-resource habitats (i.e. steppes), the increasing trend of plant diversity along the resource gradient gradually weakens as plant–plant interactions start to change from facilitative to competitive [13,38,42]. Neither the facilitative nor the competitive strategy may dominate during this transition, probably leading to low dominance of plant communities and more opportunities for plants with a ruderal strategy [43]. Therefore, the biotic drivers of the productivity–biodiversity relationship should be less pronounced, while a resource-imposed positive relationship may still exist, if resources increase both productivity and biodiversity. In high-resource habitats (i.e. meadows), both interspecific competition [44] and negative soil feedback effects [45,46] are strong, which could lead to negative productivity–biodiversity relationships [13,44,47,48]. Meanwhile, the response of productivity and biodiversity to increasing resource abundance could be asynchronous due to different limitations beyond resources. For instance, plant diversity might eventually be limited by the local species pool [49,50], whereas productivity could be limited by light [51] or temperature [17]. These uncertain direct resource effects (e.g. when resource abundance ceases to increase productivity/biodiversity, or both) are probably the reason why the shape of the productivity–biodiversity relationship varies at large scales. For example, if both productivity and biodiversity stop increasing at the high-resource end of the gradient as resource abundance increases, a unimodal relationship is expected, as plant–plant interactions change from facilitative to competitive; however, if they keep increasing along the resource gradient, the resource effects will impose a positive productivity–biodiversity relationship that could override the negative one derived from plant–plant interactions at the high-resource end, leading to a plateauing relationship (Fig. 1b). Decomposing the resource effects is therefore important for understanding why the shape of the productivity–biodiversity relationship varies at large scales. This is of particular significance under global environmental change, for example, increased nitrogen deposition [52].

Integrating all these concepts, we used data from a national survey of 97 natural grasslands across Chinese grasslands (Fig. S1) to test the following hypotheses: (i) the shape of the productivity–biodiversity relationship varies at different levels of resource abundance; and (ii) when we control for the direct resource effect on plant productivity and biodiversity, the productivity–biodiversity relationship should be unimodal across different levels of resource abundance at the large scale.

## RESULTS

To test our hypotheses, we first computed the resource diversity (RD) index to represent resource abundance gradients, and tested their correlations with above-ground live biomass (AGB) and SR across different grassland types. Then, we explored how AGB and SR change along the computed RD gradient via the decoupling index and coupling degree. As their changes were found to be asynchronous, we conducted further analyses to compare the field-observed AGB–SR relationships and the partial AGB–SR relationships, by excluding resource effects in different resource scenarios. Integrating all these results, we investigated how the relationship between AGB and SR depends on resource abundance. Moreover, changes in plant community composition and phylogenetic diversity along the RD gradient were also analyzed to provide potential explanations for changing AGB–SR relationships. More information regarding the analyses can be found in the Methods section.

### Resource diversity across Chinese grasslands

Mean annual precipitation (MAP; referring to water), total organic carbon (TOC), total nitrogen (TN) and total phosphorus (TP) were used to generate the RD index (see the Methods section for detailed explanations; results using total or available nutrients are also compared later in this section). Generally, RR, RE and RD increased from desert to steppe to meadow (Fig. S2a; significant comparisons were marked at *p* < 0.05), while AGB and SR also exhibited a similar increasing trend (Fig. S2b).

Our results suggested that RD is a better predictor of both AGB and SR than individual resources. Among all 97 grasslands, RD explained a proportion of variation in both AGB and SR comparable to a multiple regression using all four resources (R^2^ = 0.30 vs. 0.37 for AGB, R^2^ = 0.31 vs. 0.39 for SR). Moreover, while the correlation between individual resources and AGB or SR varies among different grassland types (e.g. TN had the strongest correlation with SR in meadows, whereas MAP was more influential on SR in desert grasslands; Table 1), the correlations between RD and AGB or SR were at least comparable, if not higher, in most grassland types (mostly no less than 10% of the correlation coefficient; Table 1 and S1). For instance, RD showed strong and consistent correlation with SR in both temperate and alpine grasslands (*r* = 0.62, *p* < 0.01 for both; Table S1), while it had the strongest correlation with AGB in alpine grasslands (*r* = 0.81, *p* < 0.01; Table S1).

**Table 1.** Pearson correlation matrix between AGB/SR and each resource abundance index.


AGB^a^	MAT^b^	MAP^c^	TOC^d^	TN^e^	TP^f^	NH_4_-N^g^	NO_3_-N^h^	AP^i^	RR^j^	RE^k^	RD^l^

Meadow	−0.22	0.80^**^	0.79^**^	0.64^**^	0.4	0.77^**^	0.29	0.73^**^	0.76^**^	−0.22	0.79^**^
Steppe	0.05	0.31*	0.29*	0.26*	0.25*	0.29*	0.32^**^	0.29*	0.31*	0.2	0.34^**^
Desert grassland	0.41	0.51	0.69	−0.24	0.23	−0.03	−0.26	0.43	0.56*	0.17	0.52
Overall	−0.01	0.53^**^	0.54^**^	0.47^**^	0.34^**^	0.52^**^	0.35^**^	0.43^**^	0.55^**^	0.18	0.56^**^
SR^m^	MAT	MAP	TOC	TN	TP	NH_4_-N	NO_3_-N	AP	RR	RE	RD
Meadow	0.24	0.37	0.50*	0.58*	0.36	0.38	−0.31	0.25	0.51*	−0.22	0.53*
Steppe	0.31^**^	0.46^**^	0.50^**^	0.51^**^	0.19	0.07	−0.26*	−0.09	0.47^**^	0.33^**^	0.50^**^
Desert grassland	0.66*	0.73^**^	0.13	0.1	0.05	0.25	−0.26	0.09	0.65*	0.27	0.63*
Overall	0.30^**^	0.55^**^	0.48^**^	0.55^**^	0.28^**^	0.23*	−0.11	0.05	0.48^**^	0.36^**^	0.56^**^

a AGB, above-ground biomass.

b MAT, annual mean temperature.

c MAP, annual mean precipitation.

d TOC, total organic carbon.

e TN, total nitrogen.

f TP, total phosphorus.

g NH_4_-N, ammonium nitrogen.

h NO_3_-N, nitrate-nitrogen.

i AP, plant-available phosphorus.

j RR, resource richness.

k RE, resource evenness.

l RD, resource diversity.

m SR, species richness.

* *p* < 0.05; ^**^, *p* < 0.01.

Regarding the two aspects of RD, RR was positively correlated with both AGB and SR, regardless of grassland types; while RE was only significantly positively correlated with SR in steppes and across all grasslands (Table 1). In most cases, RD had a higher correlation coefficient with both AGB and SR than RR (Table 1). Moreover, our results suggested that in habitats with similar RR, the SR of habitats with lower RE was significantly lower than that of habitats with higher RE, while the AGB is similar. For instance, seven sites in our study had higher TP but lower MAP, TOC and TN, compared with the mean values for all 97 grasslands. Compared with the other 25 sites with similar RR (0.098 ± 0.012 for the 7 sites vs. 0.104 ± 0.005 for the 25 sites, *F* = 0.27, *p* = 0.61) and much higher RE (0.670 ± 0.013 vs. 0.853 ± 0.012, *F* = 59.34, *p* < 0.001), the former had a significantly lower SR (8.43 ± 1.36 vs. 13.84 ± 0.84, *F* = 9.55, *p* < 0.01) and a slightly but non-significantly lower AGB (40.18 ± 15.19 vs. 48.38 ± 9.16, *F* = 0.18, *p* = 0.67).

We further compared the regression results for AGB and SR when using total or available nutrients. Available nutrients (ammonium, nitrate and plant-available phosphorus) were generally slightly less correlated with AGB compared with total nutrients (TOC, TN and TP), while they had almost no correlations with SR (Table 1). The correlation coefficient between SR and RD calculated from total nutrients was twice as high as calculated from available nutrients (*r* = 0.56 vs. 0.27, *p* < 0.001 for both), while similar correlations were found between AGB and RD using either total or available nutrients (*r* = 0.56 for total nutrients vs. 0.67 for available nutrients, *p* < 0.001 for both). Nevertheless, ammonium and plant-available phosphorus were significantly correlated with RD calculated from total nutrients across all grasslands (*r* = 0.64, *p* < 0.001, for ammonium; *r* = 0.46, *p* < 0.001, for plant-available phosphorus; Table S2).

### Changing AGB and SR along the RD gradient

**Figure 2.**
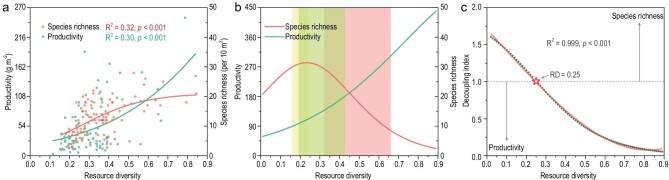
Changing effects of RD on productivity and SR. (a) Non-linear relationship between RD and productivity or SR. (b) The derivative of the two fitted functions for productivity and SR. Colored shading represents the RD range in terms of mean ± standard error (SE) of each grassland type (yellow, green and red stand for desert, steppe and meadow, respectively). (c) Decoupling of the relationship between productivity and SR along the RD gradient. The decoupling index above or below 1 indicates a relatively stronger resource effect on SR or productivity, respectively. Gray dots represent the calculated decoupling index, while the red line stands for the quadratic regression. The tipping point is marked as a red pentagram.

We first tested how AGB and SR change along the computed RD gradient. Although both increased along the RD gradient (Fig. 2a), a plateauing relationship was found between RD and SR based on model comparisons (Table S3; R^2^ = 0.33 vs. 0.31, Akaike Information Criteria (AIC) = 300.58 vs. 304.28 for the logistic and linear relationships, respectively). The decoupling changes of AGB and SR along the RD gradient were solved by using the derivative and decoupling index of the best-fitted functions. These results further supported that SR first increases with RD, peaking at medium-resource sites, and then gradually flattens (Fig. 2b). The decoupling index also revealed a decoupling of the relationship between AGB and SR along the RD gradient (Fig. 2c). The tipping point where RD became more influential on AGB than SR occurred at the intermediate resource level (RD = 0.25, which was slightly lower than its median of 0.28).

### Plant AGB–SR relationships at and across different levels of RD

**Figure 3.**
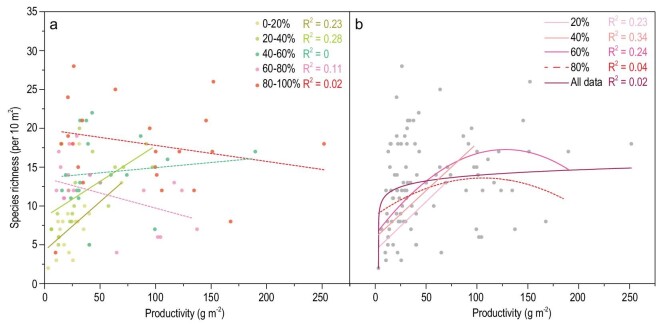
Plant productivity–richness (P–R) relationships at and across different levels of RD. (a) P–R relationships at different levels of RD. (b) P–R relationships from the least 20%, least 40%, 60%, 80%, to all (100%).

Inconsistent plant AGB–SR relationships were revealed across different levels of RD (see Methods section for details on grouping and sensitivity test). Generally, as RD increased, the AGB–SR relationship gradually changed from positive to negative, and only low-resource groups exhibited significant relationships (both positive; Fig. 3a). By incorporating more grasslands with higher RD into the group of grasslands with the lowest RD, a unimodal relationship was observed across sites with relatively low and medium resources, while such a positive relationship faded away as more high-resource grasslands were included (Fig. 3b). Nevertheless, a significant but weak linear relationship was detected with all data (R^2^ = 0.05, *p* < 0.05).

**Figure 4.**
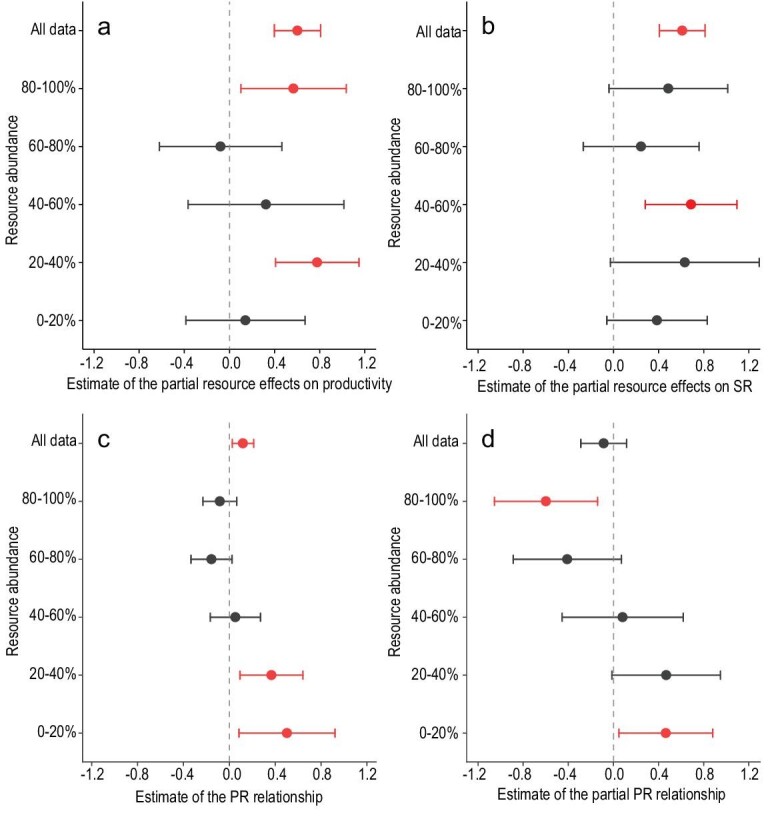
Estimated relationships among RD, plant productivity and SR across different levels of RD. (a) Partial resource effects on productivity excluding SR effects. (b) Partial resource effects on SR excluding productivity effects. (c) Observed P–R relationships. (d) Partial P–R relationships excluding resource effects. Dots represent the mean value of the model predictors (slope for sub-plots a–c; standard regression coefficient for sub-plot d), while error bars represent 95% confidence intervals. Results are considered significant if error bars do not overlap with zero and are colored red.

Overall, RD had a comparable positive partial effect on both AGB (excluding effects of SR) and SR (excluding effects of AGB) across the studied Chinese grasslands (standard regression coefficient = 0.60 vs. 0.61; Fig. 4a and b). Partial effects of RD on SR were positive across different resource levels, with most having confidence intervals above or slightly below zero (exceptions found at 60%–80% level; Fig. 4b); however, the partial effects of RD on AGB were only significantly positive at 20%–40% and 80%–100% levels (Fig. 4a). Notably, the partial resource effect size on AGB and SR was similar at the high RD level (80%–100%) (standard regression coefficient = 0.57 vs. 0.49). As RD increased, the AGB–SR relationship was significantly positive across all grasslands and was gradually changing from significantly positive to neutral to non-significantly negative (Fig. 4c). However, by excluding RD effects, changing relationships from significant positive (0%–20%) to negative (80%–100%) were found, resulting in an overall non-significant negative relationship across all sites (Fig. 4d). These partial relationships were obtained from multiple regression analysis using RD and AGB to predict SR. However, such patterns were not detected across different levels of AGB (Fig. S3). Together with the decreasing coupling degree between AGB and SR (*p* < 0.05; Fig. S4), our results showed that as RD increased, the AGB–SR relationship gradually changed from significantly positive to neutral and then to significantly negative, which resembles the pattern of the unimodal relationship.

**Figure 5.**
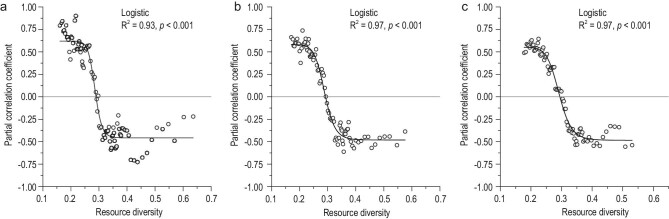
Relationships between the partial correlation coefficient and the corresponding moving average of RD. (a) Using 10 adjacent sites; (b) using 15 adjacent sites; (c) using 20 adjacent sites. For instance, the 10 adjacent sites refer to the 1 to 10, 11 to 20, etc., and 88 to 97 sites along the RD gradient.

In addition to the analyses based on predefined resource groups, a moving window method was also used to gradually detect the changing partial relationship between AGB and SR by excluding resource effects. Our results suggested that the partial AGB–SR relationship gradually changed from positive to negative along the RD gradient (Fig. 5). Furthermore, a significant positive partial effect of AGB on SR was found for the lowest 20% to 60% of sites sorted by RD, whereas a significant negative effect existed in the highest 20% to 50% of sites (Table S4). Overall, the strength of both positive and negative relationships decreased from the lowest/highest RD to the intermediate level, and finally became non-significant, which reinforces the above results of a unimodal AGB–SR relationship. Moreover, the changing AGB–SR relationship from positive to negative was generally found along different resource gradients (e.g. TOC, TN, TP, ammonium and nitrate; Fig. S5), while collectively, the trend was quite clear and robust along the RD gradient.

### Plant community change along the RD gradient

Plant community composition in terms of the four functional groups varied at different levels of RD (stress = 0.1164, R^2^ = 0.13, *p* < 0.01; Fig. S6a). The R2 group (RD ranging from 0.21 to 0.25), in which the tipping point of the changing resource effects between AGB and SR was found, had the highest proportion of ruderals (Fig. S6b and c), which was a different community composition character compared with other groups (*p* = 0.047 vs. R3; *p* < 0.01 for others; Fig. S6a). Plant community composition also differed between the lowest- and highest-resource groups (R1 vs. R5, *p* = 0.057; Fig. S6a), where R1 had a significantly higher proportion of shrubs and higher Simpson's dominance than R5 (*p* < 0.05 for shrubs; *p* < 0.01 for Simpson's dominance; Fig. S6c). No significant differences were found for other pairwise comparisons (*p* > 0.1). In general, plant community composition changed from being characterized by shrubs, to more ruderals, to more perennial herbs with increasing RD, although the relative abundance of perennial grasses and forbs was not significantly higher in the relatively low-resource groups (R4 and R5 in Fig. S6c). Moreover, Simpson's dominance decreased along the RD gradient (R^2^ = 0.19, *p* < 0.001; Fig. S6b), while a quadratic relationship was revealed between RD and phylogenetic diversity (R^2^ = 0.28, *p* < 0.001; Fig. S7).

## DISCUSSION

This study shows that, as a single collective index, RD allows for effective and ecologically relevant integration of plant-essential resources. By representing the multidimensional resource gradient with RD, we are able to explain how resource effects alter the field-observed plant productivity–biodiversity relationship under different resource scenarios. These results mark a major advance towards reconciling the long-standing debate about the shape of the productivity–biodiversity relationship by revealing its dependence on multidimensional resource gradients.

Based on well-established evidence that grassland productivity is limited by multiple resources [10,23], we expected to find that productivity increases with RD in this study. However, to the best of our knowledge, the synergetic effects of water and nutrients on biodiversity have not been previously reported in a single large-scale comparative field study, although biodiversity has been shown to decrease with increasing nutrient abundance in experimental communities [23,25] and to increase with increasing water availability in both experimental and field studies [12,24]. Our study indicates that biodiversity shows a plateauing function in response to RD. This finding is inconsistent with many field studies using biomass to represent a resource gradient, which concluded that biodiversity peaks at medium levels of RD [13,44,47]. Therefore, the inconsistent resource effects on productivity and biodiversity and their potential ecological implications are widely overlooked by using a biomass gradient only. By adopting a multidimensional resource gradient, it is found that the relationship between productivity and biodiversity is decoupled at medium levels of RD. Productivity exhibits a faster-increasing trend than biodiversity after crossing this threshold, which can be ascribed to the changing plant strategy from colonization/stress-tolerant at low resource levels where facilitative interactions are important, to competition at high resource levels where biodiversity effects may be less pronounced [13,42]. The quadratic relationship between RD and phylogenetic diversity further reinforces this shift as ecological forces change from habitat filtering to competitive exclusion [53,54]. Thus, the observed relationship between RD and biodiversity may be the result of a three-way interaction: resources increase both productivity and biodiversity, and increased productivity in turn gradually reduces biodiversity as a result of competitive exclusion [44]. Based on these insights, four types of relationships are therefore expected along the multidimensional resource gradient: (i) a positive relationship from resource effects only (mostly at low resource levels); (ii) a plateauing or (iii) a unimodal relationship depending on the relative intensity between the positive resource effects and the negative competitive exclusion effect; and (iv) a negative relationship due to competitive exclusion (mostly at high resource levels).

At low resource levels, our results showed that resources increase both productivity and biodiversity, causing a positive relationship between them. Meanwhile, the increase in productivity and biodiversity is expected to reinforce the increase in both [13,38,40,55]. We thus assume that the strong positive relationship between productivity and biodiversity found in this study results from a combination of these positive relationships. On the other hand, an imposed positive productivity–biodiversity relationship by resources is also revealed at high resource levels, whereas the presence of a negative relationship is probably due to the strong interspecific competition at high productivity [44]. As a result of the opposing effects of the imposed positive relationship and the biotic-induced negative relationship, no significant productivity–biodiversity relationship was found at high resource levels. However, no significant resource effects were found on either productivity or biodiversity at medium resource levels, or on the productivity–biodiversity relationship, leading to a non-significant observed relationship. A similar result was also reported by using a plant biomass gradient, with a much weaker relationship in the medium-biomass group compared with the low- and high-biomass groups [13]. When removing the resource effects, the changing productivity–biodiversity relationship along the RD gradient can be ascribed to shifting plant strategies as discussed above. However, our results reveal a transition of plant–plant interactions from low- to high-resource habitats, which is an extension of the transition from colonization (or stress-tolerant) to competitive strategy [42] by involving the ruderal strategy [43] in between. At medium resource levels, plant communities showed a higher dominance of a ruderal strategy. We thus speculate that no clear productivity–biodiversity relationship is observed at medium resource levels, because the populations of ruderal species are highly dynamic [43]. Taken together, studies with lower variability in RD are supposed to exhibit three types of productivity–biodiversity relationships: (i) a positive relationship in the low-resource scenario; (ii) no relationship in the medium-resource scenario; (iii) no relationship or a negative relationship in the high-resource scenario.

As for large-scale studies with broader levels of RD, unimodal relationships (also including quadratic and plateauing relationships) are more likely to occur, which is considered as an accumulation of many linear relationships from different habitats [47]. By removing the resource effects, our results revealed a gradually changing productivity–biodiversity relationship from significantly positive to significantly negative from low- to high-resource habitats, which is in line with the stress-gradient hypothesis [13,56,57], suggesting a standard unimodal relationship along the resource gradient. However, a linear rather than unimodal relationship is observed at the national scale, as the resource-induced positive productivity–biodiversity relationship masked the negative relationship arising from interspecific competitions in high-resource habitats. Therefore, the previous debates over the shape of this relationship at large scales [5–8] probably stem from two resource-related aspects. Firstly, the classical explanation (competition exclusion) for the hump-back model is originally based on the environment stress gradient rather than the productivity gradient [44]. For the unimodal productivity–biodiversity relationship, it is assumed that the productivity gradient could represent a stress gradient. This assumption might be true when a large number of samples are collected with a wide range of productivity levels, as was done in HerbDivNet [2], while it may not be the case in large-scale surveys with fewer quadrats at one site (as in our study). Therefore, the mostly unexplored relationship between the productivity gradient and environmental gradient (e.g. in terms of RD) is very likely to lead to inconsistencies among large-scale studies. Secondly, even if the two gradients are well-matched, the effects of multidimensional resource abundance can conceal the productivity–biodiversity relationship that arises from biotic interactions. By integrating all these concepts, our results demonstrate that a unimodal relationship theoretically exists. Future studies should experimentally explore the relative dominance of different mechanisms that contribute to productivity–biodiversity relationships. For instance, globally distributed coordinated experiments like Nutrient Network (NutNet) are powerful and promising initiatives to explore the generality as well as context-dependencies of ecological relationships [58].

Finally, this study aims at revealing the resource effects on the productivity–biodiversity relationship rather than on productivity or biodiversity respectively. RD was calculated from MAP and total nutrients, which are the most influential and frequently measured resources in the field. We did not involve inorganic nutrients due to two reasons: (i) they are generally slightly less correlated with productivity compared with total nutrients, and are poorly correlated with biodiversity, probably because of their pronounced temporal dynamics [32]; (ii) they are often correlated with total nutrients, especially with TOC. Generally, RD could well represent the overall effects of all selected essential resources in studying how multidimensional resource abundance affects grassland productivity and biodiversity, regardless of which resource is more important across different habitats. Moreover, the RD could help to reveal the hidden effects of resources on a plant community. For instance, in this study, no significant relationship is found between each resource and productivity in desert grassland, whereas a significantly positive resource effect on productivity is revealed by using RR, an essential aspect of RD. Moreover, consistent with our hypothesis, RE per se is only associated with biodiversity, which might be ascribed to the niche dimension hypothesis that a more balanced nutrient supply can increase the number of resource dimensions and benefit species coexistence [23,34,35]. Our results reinforce such ideas by proving that one extremely abundant resource is probably only beneficial to a few species and not others, as resource-uneven habitats have much lower biodiversity (habitats with high resource inequity) than those habitats with similar RR but much higher RE (habitats with high resource equity). Therefore, RE shows potential as a promising measurement of the resource dimension in field studies. Taken together, the RD index has two advantages over a single resource dimension or principal component analysis (PCA) in large-scale studies of resource effects on productivity–biodiversity relationships. First, it captures well the resource effects, although the relative contribution of different resources always changes in large-scale studies due to changing types of resource limitations [26], in which case considering a single resource might only lead to inaccurate conclusions. Second, it considers the evenness among resources, an important factor for plant SR, which is neglected in other methods such as the PCA. Nevertheless, we are aware that the selection of resources in computing RD may depend on the targeted ecosystem properties (e.g. plant or microbial biodiversity), which should be carefully considered in the application of this index.

## CONCLUSIONS

By establishing RD as an innovative collective index, the present study is able to isolate the biotically induced productivity–biodiversity relationship by excluding the direct effects of resources on plant communities. Our results indicate that previous controversy may be partly due to neglecting the separation of resource gradients from productivity gradients at large scales, thus providing new insights into the long-standing debate over productivity–biodiversity relationships. Yet, environmental variables other than resources (e.g. soil pH) might affect productivity–biodiversity relationships through their direct influences on productivity and biodiversity or their control over the resource availability, which warrants further studies. Overall, the present study suggests that it is important to consider both the resource effects on plant productivity and biodiversity, as well as changing plant–plant interactions along multidimensional resource gradients, to better understand plant community dynamics in a changing world.

## METHODS

### Study area

Being part of the Eurasian steppe, Chinese grasslands were chosen for our study as they cover a wide range of climatic conditions and soil types, as well as diverse grassland ecosystems. Therefore, it is an ideal region for exploring the resource effects on productivity–biodiversity relationships. Our sampling sites stretched all over Chinese grasslands, from the eastern Mongolian Plateau to the western Tibetan Plateau. In total, we collected data from 97 sampling sites (i.e. 14 desert grasslands, 37 temperate steppes, 30 alpine steppes and 16 meadows), including soil properties (TOC, TN, TP and inorganic nutrients), plant community composition, and plant SR and AGB at peak biomass (Fig. S1). Generally, the larger the distribution area of a given grassland type is [59], the more sampling sites were included. Based on data from 1980 to 2014 from China Meteorological Data Service Center (http://data.cma.cn/en), the mean annual temperature (MAT) and MAP for desert, temperate steppe, alpine steppe and meadow are 0.86°C/216.70 mm, 2.60°C/311.79 mm, −1.97°C/282.32 mm and 0.63°C/393.60 mm, respectively (Table S5). Temperate and alpine steppes share similar resource conditions (Table S5); therefore, desert, steppe and meadow are roughly considered as low-, medium- and high-resource habitats, respectively. However, as multidimensional resource abundances can largely vary even within the same grassland type, our analyses are based on the calculated RD gradient rather than grassland types.

### Field sampling and measurements

All sampling sites are tens of kilometers apart. Previous studies suggested that the studied grasslands are highly spatially heterogeneous—the spatial autocorrelation in soils and plant communities usually exists within a few meters [60]. Therefore, these sites are considered to be independent of each other. At each sampling site, a 10 m × 10 m sampling plot was established at a relatively homogeneous area without anthropogenic disturbances such as grazing in the sampling year, and was at least 500 m away from highways to exclude potential disturbances caused by traffic [61]. Five quadrats (1 m × 1 m for desert and steppe; 0.5 m × 0.5 m for meadow) were selected and investigated in the center and at the four corners of a 10 m × 10 m sampling plot [62,63]. Species that were not found in all five quadrats but existed in the 10 m × 10 m sampling plot were also recorded for further analyses. Above-ground vegetation was cut to the ground and sorted to species at peak biomass, oven-dried at 65ºC for 72 h, and then weighed for dry biomass assessments. Topsoil samples (0–5 cm from the surface) were collected with a 7 cm auger in each quadrat after removing stones, roots and micro-arthropods by sieving (at 2 mm), then sealed in plastic bags and stored at −20ºC until further analysis. We used SR at a site (the total number of species existing in the 10 m × 10 m plot) to characterize biodiversity, and the AGB per square meter from all five quadrats to represent productivity. Thus, we used the AGB–SR relationship for the study of the grassland productivity–biodiversity relationship.

Soil TOC was measured by a TOC Analyzer (Liqui TOC II; Elementar Analysensysteme GmbH, Hanau, Germany). Soil TN was measured on an auto-analyzer (SEAL Analytical GmbH, Norderstedt, Germany) using the Kjeldahl method [64]. Soil TP was measured after wet digestion with H_2_SO_4_ and HClO_4_ by a Ultraviolet–visible spectrophotometer (UV2700, SHIMADZU, Japan). Ammonium and nitrate nitrogen were extracted with 2 M KCl while plant-available Olsen P was extracted with 0.5 M NaHCO_3_, then measured by the colorimetric method with a Smart Chem 200 Discrete Auto Analyzer (AMS, Italy). Site averages of all corresponding data collected from five quadrats were used for further statistical analyses (i.e. the relative abundance of each plant species, AGB and soil nutrients).

### RD gradient

We used an index called ‘resource diversity’ to represent the multidimensional resource gradient in grasslands based on Liu *et al.* [32]. Water (represented using MAP) and three main macronutrients (TOC for carbon, TN for nitrogen and TP for phosphorus) were used to compute RD due to their well-known role in driving biodiversity and productivity [65–68]. TOC was selected because many soil inorganic nutrients, such as inorganic P, Cu, Zn, Fe and Mn, are strongly associated with it [67,69]. These macronutrients represent the soil fertility at a site, which is consistent with our idea to create a multidimensional resource abundance gradient. MAT was not used, because it is not a resource that plants can utilize directly. Furthermore, we used MAP and inorganic nutrients (i.e. ammonium, nitrate nitrogen and plant-available Olsen P) to compute another RD index and then compared its correlations with AGB and SR with the former index using macronutrients.

Resource diversity was calculated based on an improved radar chart method [32] (Fig. S8). However, a minor modification to the standardized method had been made. Briefly, the relative abundances of each essential resource across all sampling sites were calculated by the Z-score method and all converted to positive values by adding 2 (the smallest integer). At each sampling site, the standardized relative abundance of each essential resource was used as the radius of one sector, together, building up a radar chart with four sectors (Fig. S8). RR and RE at a given location were then computed based on the total area (S) and the perimeter (L) of all sectors following Liu *et al*. [32]:

(1) \begin{eqnarray*}{S}_i = \sum\limits_{j = 1}^n {{S}_j = } \sum\limits_{j = 1}^n {\pi {r}_j^2\quad j = 1,2, \ldots ,n} \end{eqnarray*}

(2) \begin{eqnarray*} {L}_i &=& \sum\limits_{j = 1}^n {L}_j = 2\left| {{r}_{\max } - {r}_{\min }} \right|\nonumber\\ && +\, \sum\limits_{j = 1}^n {2\pi {r_j} \quad j = 1,2, \cdots ,n} \end{eqnarray*}

(3) \begin{eqnarray*}R{R}_i = {S}_i/max{S}_i.\end{eqnarray*}

(4) \begin{eqnarray*}R{E}_i = {S}_i/\left[ {\pi {{\left( {{L}_i/2\pi } \right)}}^2} \right] = 4\pi {S}_i/{L}_i^2.\end{eqnarray*}

Here, *i* stands for the different sites; *n* represents the number of essential resources for plant communities, which is four in this study; *r_max_* and *r_min_* represent the maximum and minimum relative abundance of the four resources; and *r_j_*stands for the relative abundance of the *j^th^* resource.

Generally, RR is defined as the average of the relative abundance of all selected essential resources. It is normalized to 0–1 by being divided by its max value across all sites. RE is the ratio between the total area of the radar chart formed by all selected essential resources and the area of a circle with the same perimeter (the evenest distribution of all selected essential resources), which decreases as unevenness among all selected essential resources increases. Notably, the doubled value of the difference between *r_max_* and *r_min_* refers to the part of the perimeter other than the total length of all arcs (the total length of all lines between two adjacent arcs; Fig. S8).

RD was calculated as the geometric mean of RR and RE, to reduce the potential overestimation of resource availability by using RR alone. Weight for different resources was not considered, because this study aimed to investigate the effect of RD on the AGB–SR relationship rather than AGB or SR itself. When focusing on AGB or SR themselves, the weight can be set based on a variety of methods (e.g. based on the ratios of coefficients in the multiple regression models). However, as the relative effects of each resource on AGB or SR are quite different (see Results section for details), it is not suitable or even possible to set the weight for the AGB–SR relationship.

Based on the wide range of RD, all data were classified from low to high by RD and then divided into five groups in a roughly equal manner (19 for the first four subgroups and 21 for the last one) to create resource levels ranging at the lowest 20%, 20%–40%, 40%–60%, 60%–80% and 80%–100%. We used five groups instead of the three shown in Fig. 1 to assess potential shifts in plant AGB–SR relationships along the RD gradient. Nevertheless, to test the sensitivity of grouping on our results, we used more groups starting from the lowest 20% sites, to the lowest 30%, then to the lowest 40%, etc., and eventually to all sites to explore whether the positive AGB–SR relationship widely exists in low-resource habitats and gradually fades out along the RD gradient; conversely, tests on whether the negative relationship widely exists in high-resource habitats were carried out from the group of the highest 20% sites to all sites.

Additionally, the partial correlation coefficient between AGB and SR (excluding the effect of RD) was gradually calculated along the RD gradients via a moving window method [48], providing comparable results of the changing AGB–SR relationships that were not based on the predefined resource groups. Similar to the moving average, we gradually calculated the partial correlation coefficient between AGB and SR within the 10 to 20 adjacent sites from the lowest RD to the highest RD, and plotted them against the corresponding moving average of RD. The relationships between the partial correlation coefficient and the moving average of RD were quite similar, independent of how many adjacent sites were used. Therefore, we only presented results using 10, 15 and 20 adjacent sites.

### Statistical analyses

The differences of RR, RE, RD, AGB and SR among the four grassland types were analyzed by one-way analysis of variance (ANOVA), and the statistical significance between groups was based on *p* values from the least significant differences (LSD) test. Linear regression was generally used in fitting the RD–AGB and RD–SR relationships within each grassland type, whereas peak function (i.e. logistic) was also used for the same variables but along the RD gradient to test our hypothesis (Fig. 1). Linear, quadratic and unimodal functions were fitted for the AGB–SR relationships across different levels of RD. Model comparisons were made based on the explained variances and the significance of the corresponding coefficients (slope for linear regression, quadratic term for quadratic regression and k for logistic regression). The observed AGB–SR relationship within each resource subgroup was calculated and presented by the slope of the corresponding linear regression. The partial effect size of RD on AGB (excluding SR effects) and SR (excluding AGB effects), and the partial AGB–SR relationship (excluding resource effects) were calculated and presented by the standard regression coefficient from multiple regression. All statistical significances were set at *p* < 0.05. All statistical tests above were performed in SPSS v. 27 (IBM Corp., Armonk, NY, USA).

### Coupling and decoupling degree

The derivative of fitted AGB–SR functions, decoupling index (DI) and coupling degree (CD) were used to investigate the asynchronous change of AGB and SR along the RD gradient. Coupling and decoupling describe the interactions among two or more systems. They are originally used in physics, then applied in studies of climate change and economy–environment interactions [70]. The DI was calculated from the fitted function to investigate the decoupling of the relationship between AGB and SR along the RD gradient. DI refers to the ratio of the changing rate per unit increasing RD of the fitted S-shape functions for SR and AGB (DI = *ΔSR_RD_/ΔP_RD_*). With the increase of RD, DI is higher than 1 when the changing rate of SR is higher than that of AGB, and it ranges from 0 to 1 when the situation reverses.

The method used in computing DI is based on continuous functions of SR and AGB (fitted curve); thus, the interactions among raw data cannot be explored. Therefore, the coupling degree was introduced to study the coupling relationship between SR and AGB with raw data. It was calculated following Lu *et al*. [70]:

(5) \begin{eqnarray*} C = {\{ S{{{R}}}_i \times {P}_i/{\left[ {(S{R}_i + {P}_i)/2} \right]}^2\} }^{1/2}, \end{eqnarray*}

where C is the coupling degree between SR and AGB, and *SR_i_* and *P_i_*represent the pairwise values of SR and AGB along the RD gradient, respectively.

### Plant community composition

This part of analysis aims at providing evidence for the assumed changing plant–plant interactions along the RD gradient. No species-sorted biomass data were available at six sampling sites, so 91 sites were used for the analysis of plant community composition. All plant species were sorted into four functional groups, namely, perennial grasses, perennial forbs, ruderals (annual and biennial grasses and forbs) and shrubs. Then, the relative abundance of each functional group at a site was calculated. Based on Grime's Three Primary Strategies [43], perennial grasses and forbs are characterized by the competitive strategy, ruderals represent the ruderal strategy, and shrubs stand for the stress-tolerant strategy as most shrubs in our study were *Artemisia*, which is drought tolerant [71–73]. Therefore, the present study was able to explore the change in plant strategy along the RD gradient. Only eight sites have unidentified species, whose relative abundance ranges from 0.002 to 0.079 with a mean value of 0.032. The non-metric multidimensional scale analysis (NMDS) was performed to explore the differences in the composition of the four functional groups across different levels of RD. The Adonis test was used to reveal statistical significance. One-way ANOVA with the Tukey Honestly Significant Difference (HSD) test was used to explore the differences in the relative abundance of each functional group and Simpson's dominance across different levels of RD. Data sets were transformed by using square root before the ANOVA test. All related analyses for plant community composition were performed in R × 64 3.5.0 with the package of vegan [74]. Additionally, phylogenetic diversity was also calculated using Phylocom [75].

## Supplementary Material

nwac165_Supplemental_Files
