# Supplementary material for: Unimodal productivity–biodiversity relationship along the gradient of multidimensional resources across Chinese grasslands
Source: Natl Sci Rev. 2022 Aug 18;9(12):nwac165. doi: 10.1093/nsr/nwac165 (PMC9743175; doi:10.1093/nsr/nwac165)
Supplement: nwac165_Supplemental_Files [file nwac165_supplemental_files.zip › Supplymentary_information_R2.docx]

**Supplementary information for**

**Unimodal productivity-biodiversity relationship along the gradient of multidimensional resources across Chinese grasslands**

Yanfen Wang, Jianqing Du, Zhe Pang, Yali Liu, Kai Xue, Yann Hautier, Biao Zhang, Li Tang, Lili Jiang, Baoming Ji, Xingliang Xu, Jing Zhang, Ronghai Hu, Shutong Zhou, Fang Wang, Rongxiao Che, Di Wang, Chaoting Zhou, Xiaoyong Cui, Nico Eisenhauer & Yanbin Hao

*Correspondence to: yfwang@ucas.ac.cn.


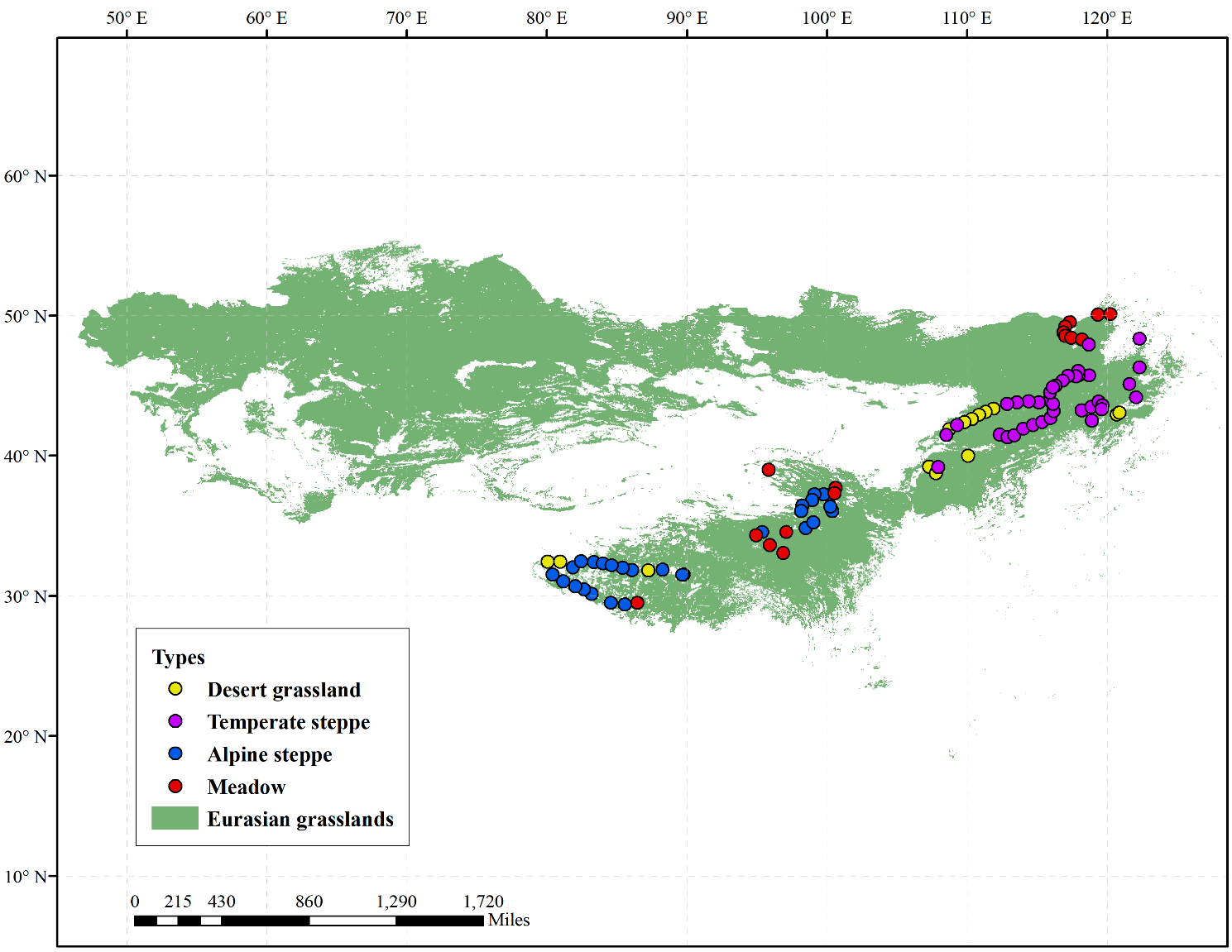


**Figure S1.** Site locations. In total, 97 sampling locations were used in the present study. Different colors are used to present different grassland types.


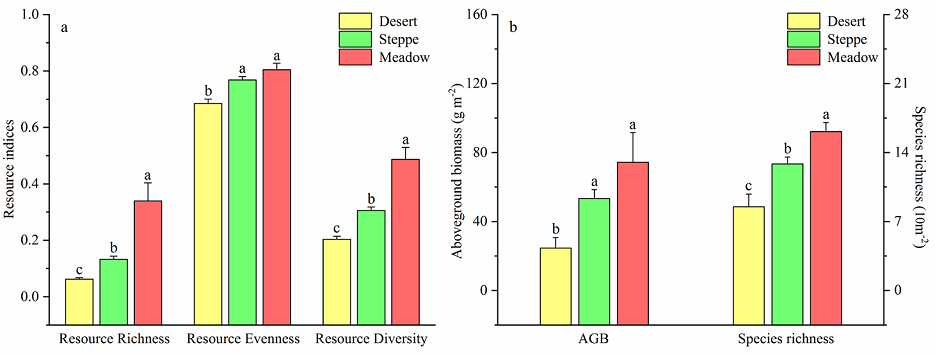


**Figure S2.** Differences in resource indices, aboveground biomass (AGB), and species richness across four grassland types. (a) differences in resource indices; (b) differences in AGB and species richness. Letters a, b, and c represent significant differences at *p* < 0.05. The histogram with an error bar represents the mean value of each index ± standard error of the mean (SEM). There are 14 desert grasslands, 67 steppes, and 16 meadows.


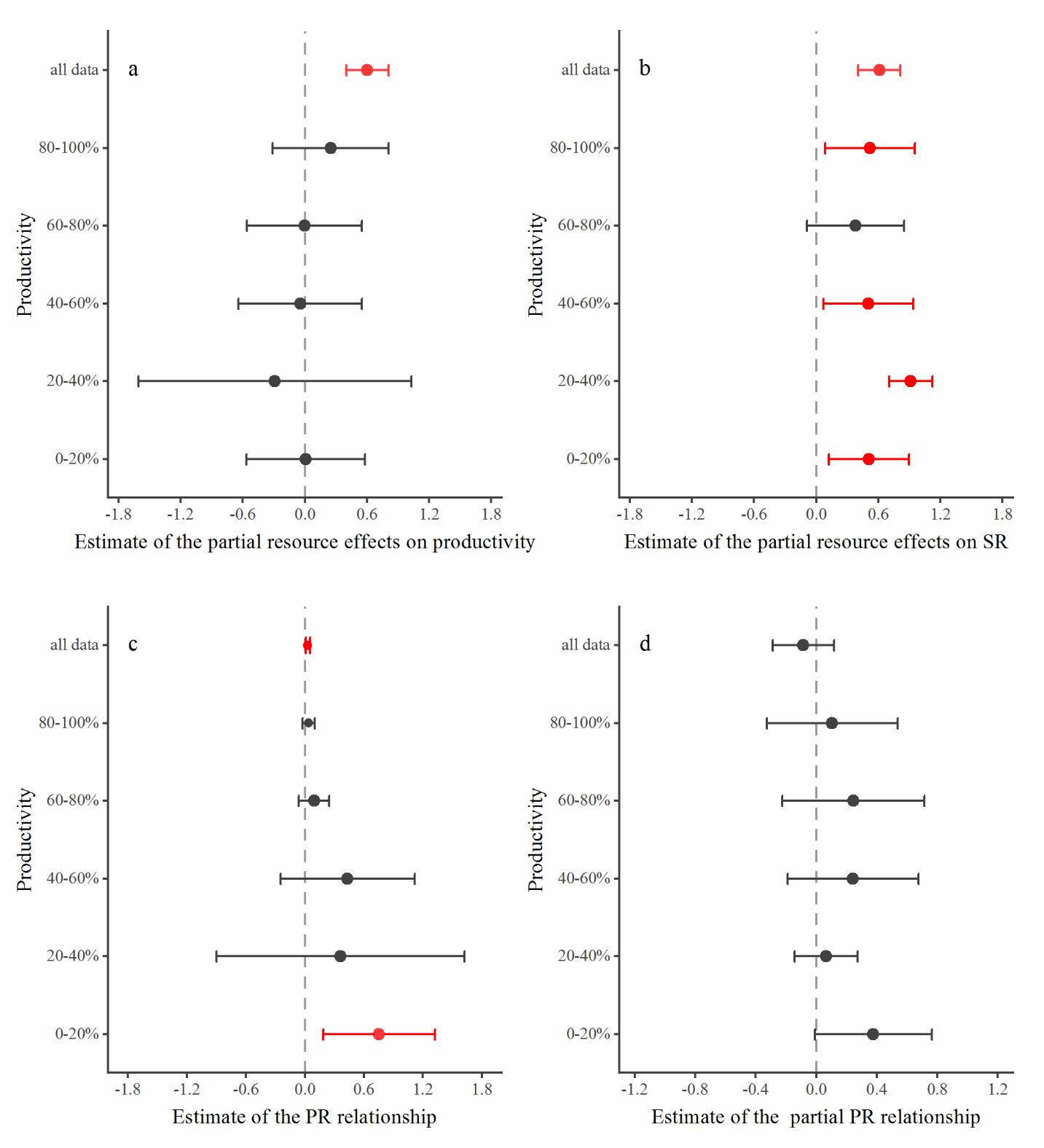


**Figure S3.** Plots showing the estimated relationships among resource diversity, productivity, and species richness (SR) across different levels of productivity. (a) partial resource effects on productivity excluding SR effects; (b) partial resource effects on SR excluding productivity effects; (c) observed productivity-richness (PR) relationships; (d) partial productivity-richness relationships excluding resource effects. Dots represent the model predictors (slope for sub-plots a-c; standard regression coefficient for sub-plot (d), while error bars represent the range of 95% confidence intervals. Results are considered significant if error bars do not overlap with zero and are colored red.





**Figure S4.** Productivity and species richness relationship along the resource gradient. The grey dots represent the coupling degree between productivity and species richness along the resource gradient (left y-axis). The blue dot with an extended line presents the standard regression coefficient between productivity and species richness with the range of 95% confidence intervals in each resource group from the most resource-poor 20% to the most resource-rich 20%. Dots are marked solid for significant relationships. For better visualization, negative relationships are presented with absolute values.





**Figure S5.** Relationships between the partial correlation coefficient and the corresponding moving average of different resources using 20 adjacent sites.


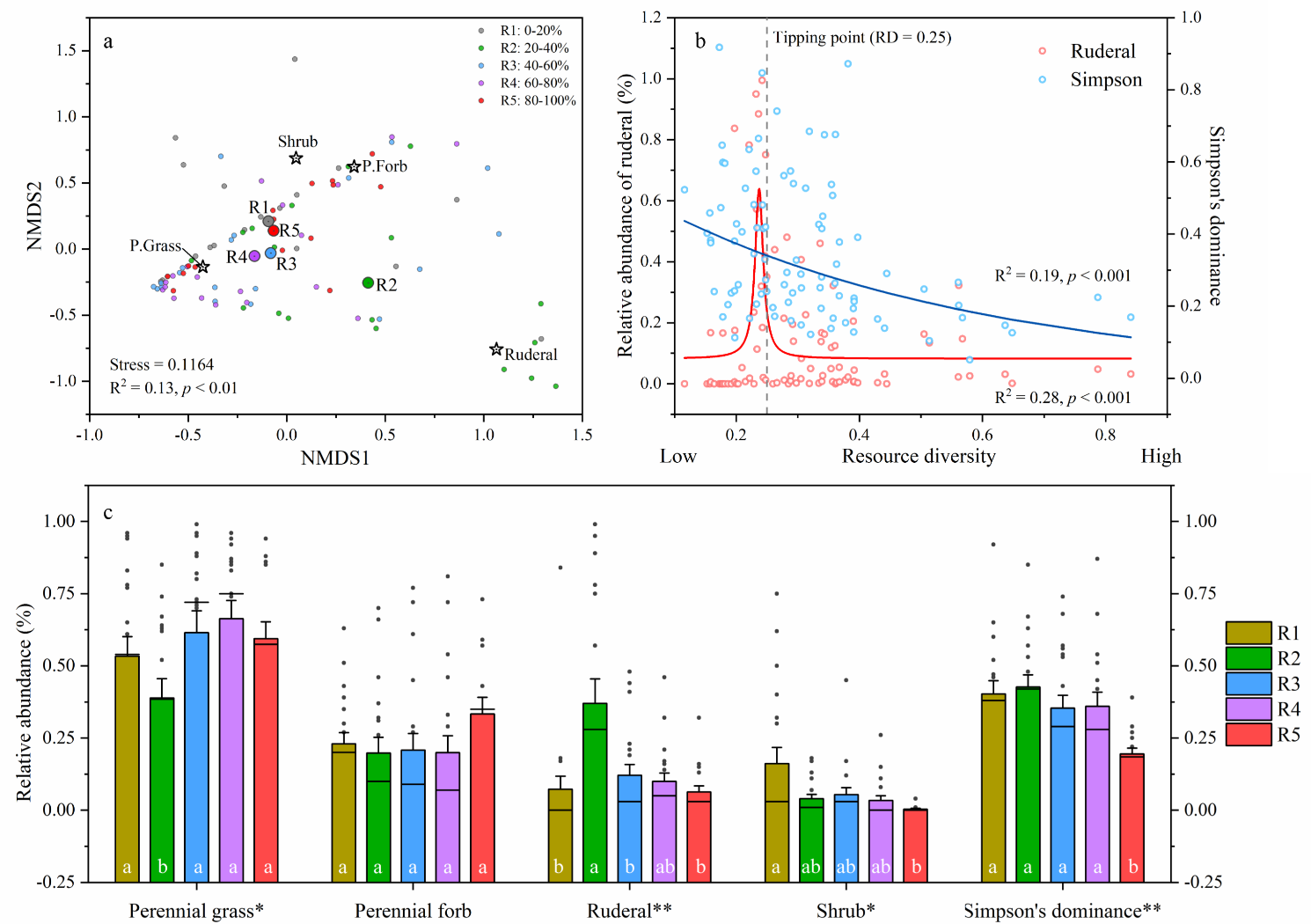


**Figure S6.** Change of plant community composition along the resource gradient. (a) cluster analysis of plant community composition in terms of functional groups; (b) change of the relative abundance of ruderal and Simpson’s dominance along the resource gradient; (c) differences in the relative abundance of each functional group and Simpson’s dominance across different levels of resource diversity. R1 to R5 represent the most resource-poor 20% group to the most resource-rich 20% group, respectively. The large dots with the label “R1” to “R5” visualize the centroids of the corresponding resource group, while the group differences are based on Adonis analysis. The grey pentagrams represent the four functional groups, namely, P. Grass for perennial grasses, P. Forb for perennial forbs, and Ruderal for the annual and biennial plants. In subplot c, * and ** indicate significant differences among groups at *p* < 0.05 and *p* < 0.01, respectively; letters a and b are used to mark significant pairwise differences at *p* < 0.05; the histogram with error bars represents the mean value ± standard error of the mean (SEM), while the black lines in or outside of the histogram indicate the median.





**Figure S7.** Relationship between resource diversity and plant phylogenetic diversity.


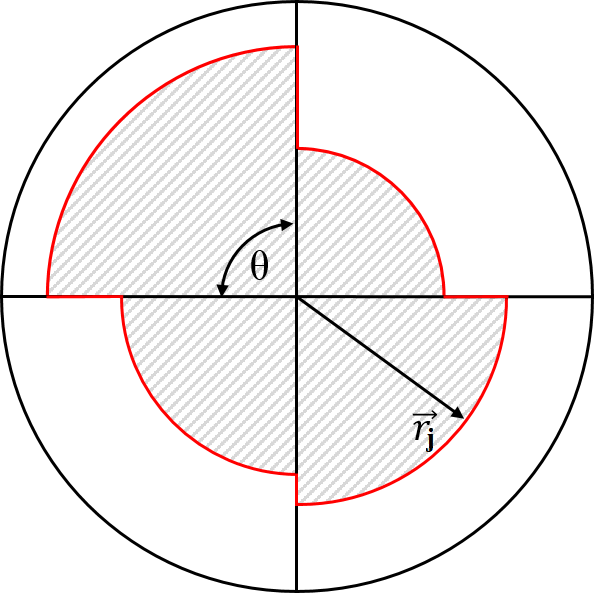


**Figure S8.** The radar chart method. *r_j_* stands for the relative abundance of the jth resource. θ represents the weight of each resource which refers to *f_j_* in equations (1) and (2). The area of each sector refers to *S_j_* in equation (1). The red line presents the total perimeter of all sectors which refers to *L_i_* in equation (2).

**Table S1.** Pearson correlation matrix between AGB/SR and each resource abundance index in temperate and alpine grasslands.

| AGB | MAT | MAP | TOC | TN | TP | NH_4_-N | NO_3_-N | AP | RR | RE | RD |
| --- | --- | --- | --- | --- | --- | --- | --- | --- | --- | --- | --- |
| Alpine grassland | 0.67** | 0.70** | 0.71** | 0.67** | 0.78** | 0.59** | 0.28** | 0.60** | 0.74** | 0.37* | 0.81** |
| Temperate grassland | 0.16 | 0.16 | -0.11 | -0.09 | -0.16 | -0.09 | -0.02 | -0.06 | -0.07 | 0.13 | -0.05 |
| Overall | -0.01 | 0.53** | 0.54** | 0.47** | 0.34** | 0.52** | 0.35** | 0.43** | 0.55** | 0.18 | 0.56** |
| SR | MAT | MAP | TOC | TN | TP | NH_4_-N | NO_3_-N | AP | RR | RE | RD |
| Alpine grassland | 0.11 | 0.53** | 0.60** | 0.65** | 0.39* | 0.40* | -0.04 | 0.25 | 0.58** | 0.25 | 0.62** |
| Temperate grassland | 0.70** | 0.70** | 0.60** | 0.60** | 0.14 | 0.33* | 0.16 | -0.15 | 0.56** | 0.42** | 0.62** |
| Overall | 0.30** | 0.55** | 0.48** | 0.55** | 0.28** | 0.23* | -0.11 | 0.05 | 0.48** | 0.36** | 0.56** |

AGB, aboveground biomass; SR, species richness; MAT, annual mean temperature; MAP, annual mean precipitation; TOC, total organic carbon; TN, total nitrogen; TP, total phosphorus; NH_4_-N, ammonium nitrogen; NO_3_-N, nitrate-nitrogen; AP, plant-available phosphorus; RR, resource richness; RE, resource evenness; RD, resource diversity. *, *p* < 0.05; **, *p* < 0.01.

**Table S2.** Pearson correlation matrix between major inorganic nutrients and other resource abundance index.

| All grasslands | MAT | MAP | TOC | TN | TP | RR | RE | RD |
| --- | --- | --- | --- | --- | --- | --- | --- | --- |
| NH_4_-N | 0.01 | 0.48** | 0.81** | 0.67** | 0.29** | 0.80** | -0.01 | 0.64** |
| NO_3_-N | -0.33** | 0.10 | 0.20 | 0.22* | 0.15 | 0.13 | 0.05 | 0.20 |
| AP | -0.07 | 0.37** | 0.42** | 0.39** | 0.41** | 0.48** | 0.03 | 0.46** |

AGB, aboveground biomass; SR, species richness; MAT, annual mean temperature; MAP, annual mean precipitation; TOC, total organic carbon; TN, total nitrogen; TP, total phosphorus; NH_4_-N, ammonium nitrogen; NO_3_-N, nitrate-nitrogen; AP, plant-available phosphorus; RR, resource richness; RE, resource evenness; RD, resource diversity. *, *p* < 0.05; **, *p* < 0.01.

**Table S3.** Model comparisons.

| Figure | Resource  levels | Fitting types | N | df | adjusted R^2^ | k/QT/Slope | t | *p*  (coefficient) | F | *p*  (model) |
| --- | --- | --- | --- | --- | --- | --- | --- | --- | --- | --- |
| Fig.2a.  productivity | All | SLogistic* | 97 | 94 | 0.302 | 3.033 | 2.048 | 0.043 | 71.723 | <0.001 |
|  |  | quadratic | 97 | 94 | 0.301 | 158.62581 | 1.137 | 0.258 | 21.705 | <0.001 |
|  |  | linear | 97 | 95 | 0.299 | 190.410 | 6.480 | <0.001 | 41.987 | <0.001 |
| Fig.2a.  species richness | All | Michaelis-Menten* | 97 | 94 | 0.333 | 0.546 | 2.641 | 0.009 | 396.140 | <0.001 |
|  |  | quadratic | 97 | 94 | 0.326 | -30.900 | -1.892 | 0.062 | 24.185 | <0.001 |
|  |  | linear | 97 | 95 | 0.307 | 22.981 | 6.603 | <0.001 | 43.605 | <0.001 |
| Fig.3a. | 0-20% | quadratic | 19 | 16 | 0.187 | -0.005 | -0.235 | 0.818 | 3.072 | 0.074 |
|  |  | linear* | 19 | 17 | 0.232 | 0.126 | 2.539 | 0.021 | 6.447 | 0.021 |
|  | 20-40% | quadratic | 19 | 16 | 0.284 | -0.001 | -1.062 | 0.304 | 4.563 | 0.027 |
|  |  | linear* | 19 | 17 | 0.278 | 0.092 | 2.818 | 0.012 | 7.938 | 0.012 |
|  | 40-60% | quadratic | 19 | 16 | -0.108 | 0.0003 | 0.063 | 0.950 | 0.125 | 0.883 |
|  |  | linear* | 19 | 17 | -0.042 | 0.013 | 0.512 | 0.615 | 0.262 | 0.615 |
|  | 60-80% | quadratic | 19 | 16 | 0.048 | 0.0001 | 0.148 | 0.885 | 1.450 | 0.264 |
|  |  | linear* | 19 | 17 | 0.102 | -0.038 | -1.748 | 0.099 | 3.055 | 0.099 |
|  | 80-100% | quadratic | 21 | 18 | 0.002 | 0.002 | 0.830 | 0.418 | 1.022 | 0.380 |
|  |  | linear* | 21 | 19 | 0.019 | -0.021 | -1.174 | 0.255 | 1.379 | 0.255 |
| Fig.3b. | 0-20% | quadratic | 19 | 16 | 0.187 | -0.005 | -0.235 | 0.818 | 3.072 | 0.074 |
|  |  | linear* | 19 | 17 | 0.232 | 0.126 | 2.539 | 0.021 | 6.447 | 0.021 |
|  | 0-40% | quadratic | 38 | 35 | 0.332 | -0.001 | -0.908 | 0.370 | 10.21 | <0.001 |
|  |  | linear* | 38 | 36 | 0.336 | 0.122 | 4.438 | <0.001 | 19.692 | <0.001 |
|  | 0-60% | quadratic* | 57 | 54 | 0.241 | -0.001 | -2.254 | 0.028 | 9.885 | <0.001 |
|  |  | linear | 57 | 55 | 0.185 | 0.071 | 3.698 | <0.001 | 13.677 | <0.001 |
|  | 0-80% | quadratic* | 76 | 73 | 0.045 | -0.0004 | -1.424 | 0.159 | 2.752 | 0.070 |
|  |  | linear | 76 | 74 | 0.031 | 0.029 | 1.852 | 0.068 | 3.429 | 0.068 |
|  | 0-100% | Log* | 97 | 95 | 0.102 | - | - | - | 281.778 | <0.001 |
|  |  | quadratic | 97 | 94 | 0.051 | -0.0002 | -0.917 | 0.362 | 3.602 | 0.031 |
|  |  | linear | 97 | 95 | 0.053 | 0.030 | 2.525 | 0.013 | 6.375 | 0.013 |
| Fig.3c. | 0-20%  &  80-100% | Log* | 40 | 38 | 0.206 | - | - | - | 92.997 | <0.001 |
|  |  | quadratic | 40 | 37 | 0.138 | -0.0004 | -1.352 | 0.185 | 4.128 | 0.024 |
|  |  | linear | 40 | 38 | 0.119 | 0.046 | 2.508 | 0.017 | 6.293 | 0.017 |
|  | 20-80% | GaussMod* | 57 | 52 | 0.046 | - | - | - | 92.435 | <0.001 |
|  |  | quadratic | 57 | 54 | -0.029 | -0.0002 | -0.533 | 0.596 | 0.212 | 0.809 |
|  |  | linear | 57 | 55 | -0.016 | 0.006 | 0.378 | 0.707 | 0.143 | 0.707 |

QT = quadratic term. * indicates the model chosen to be shown in the manuscript.

**Table S4.** Changing partial productivity-richness relationships from low- to high-resource habitats and from high- to low-resource habitats.

| From low to high | Lowest 20% | Lowest 30% | Lowest 40% | Lowest 50% | Lowest 60% | Lowest 70% | Lowest 80% | Lowest 90% | All data |
| --- | --- | --- | --- | --- | --- | --- | --- | --- | --- |
| standard regression coefficient | **0.412** | **0.479** | **0.445** | **0.287** | **0.244** | 0.18 | 0.062 | -0.054 | -0.088 |
| *p* | **0.041** | **0.002** | **0.001** | **0.011** | **0.033** | 0.126 | 0.57 | 0.582 | 0.392 |
| From high to low | Highest 20% | Highest 30% | Highest 40% | Highest 50% | Highest 60% | Highest 70% | Highest 80% | Highest 90% | All data |
| standard regression coefficient | **-0.581** | **-0.456** | **-0.445** | **-0.433** | -0.235 | -0.181 | -0.123 | -0.104 | -0.088 |
| *p* | **0.017** | **0.013** | **0.004** | **0.002** | 0.08 | 0.154 | 0.297 | 0.339 | 0.392 |

Partial productivity-richness relationships refer to the partial effect of productivity on richness free from the resource effects. Significant relationships are marked in bold at *p* < 0.05.

**Table S5.** Overview of grasslands involved in the present study.

|  | No. of sites | Altitude (m) | Longitude (°E) | Latitude (°N) | MAT  (°C) | MAP  (mm) | TOC  (g kg^-1^) | TN  (g kg^-1^) | TP  (g kg^-1^) | SR  (10m^-2^) | AGB  (g m^-2^) |
| --- | --- | --- | --- | --- | --- | --- | --- | --- | --- | --- | --- |
| Overall | 97 | 40-4891 | 80.0-122.3 | 29.4-50.1 | 0.6 ± 2.6 | 302 ± 110 | 18.0 ± 19.1 | 1.7 ± 1.4 | 0.4 ± 0.2 | 13 ± 6 | 53 ± 47 |
| Desert grassland | 14 | 210-4567 | 80.0-120.8 | 31.8-43.4 | 0.9 ± 2.1 | 217 ± 84 | 4.7 ± 2.0 | 0.5 ± 0.2 | 0.3 ± 0.1 | 9 ± 5 | 25 ± 22 |
| Steppe | 67 | 40-4891 | 80.4-122.3 | 29.4-48.4 | 0.6 ± 2.6 | 299 ± 93 | 14.4 ± 10.3 | 1.5 ± 1.0 | 0.4 ± 0.2 | 13 ± 6 | 53 ± 42 |
| Temperate steppe | 37 | 40-1760 | 107.9-122.3 | 39.2-48.4 | 2.6 ± 0.6 | 312 ± 68 | 14.3 ± 8.9 | 1.5 ± 0.8 | 0.4 ± 0.2 | 14 ± 5 | 46 ± 38 |
| Alpine steppe | 30 | 2992-4891 | 80.4-100.4 | 29.4-37.3 | -2.0 ± 1.8 | 282 ± 114 | 14.7 ± 11.7 | 1.5 ± 1.1 | 0.3 ± 0.2 | 11 ± 6 | 62 ± 45 |
| Meadow | 16 | 460-4501 | 86.5-120.2 | 29.5-50.1 | 0.6 ± 2.8 | 394 ± 126 | 44.6 ± 29.3 | 3.4 ± 1.7 | 0.5 ± 0.2 | 16 ± 3 | 74 ± 66 |

Data for MAT to AGB are shown as mean ± standard error of the mean (SEM). MAT, mean annual temperature; MAP, mean annual precipitation; TOC, total organic carbon; TN, total nitrogen; TP, total phosphorus; SR, species richness; AGB, aboveground biomass.
